# 

**Published:** 1897-05-29

**Authors:** 


### EARLY EDITION.

**Notices of Births, Deaths, and Marriages** are inserted in this column at a charge of 2s. 6d. for an announcement not exceeding four lines.

**MARRIAGE.**

On the 22nd inst., at the Free Christian Church, Croydon, by the Revd. P. H. Wicksteed, M.A., Victor, youngest son of Hamel Smith, of 112, Fenchurch Street, London, and Trinidad, W.I., to May Matthews, eldest daughter of T. Fred Wicksteed, late of South Australia. (1,185)

### NOTICE TO CORRESPONDENTS.

All MS., Letters, Books for Review, and other matters intended for the Editor should be addressed The Editor, "The Hospital," The Hospital Building, 28 & 29, Southampton Street, Strand, W.C.

The Editor cannot undertake to return rejected MS., even when accompanied by stamped directed envelope.

**Notice to Advertisers and Subscribers.**

All Advertisements, Orders for Copies of The Hospital, and Business Communications should be addressed to **THE MANAGER, (Not to The Editor)**, The Hospital, The Hospital Building, 28 & 29, Southampton Street, Strand, London, W.C.

The Cost of Subscription, post free, is as follows:—Per week, 2½d.; per quarter, 2s. 8d.; per half-year, 5s. 3d.; per annum, 10s. 6d. Foreign:—Per annum, 15s. 2d.; payable, in all cases, in advance.

### BOOKS RECEIVED.

H. K. Lewis.

"Notes on Medical Nursing." By the late James Anderson, M.D. Edited by Ethel F. Lamport.

The F. A. Davis Company.

"Diseases of the Ear, Nose, and Throat." By Seth Scott Bishop, M.D.

**Periodicals and Pamphlets Received.**—The Scalpel, Charity Organisation Review, Literary Digest, Medical Record, Industries and Iron, Electrical Review, Medical Pioneer, London, Medical Press, New Saturday, "Society for the Protection of Birds," "Educational Leaflets" (Part I.), "Natural Religion," "A Protest against the Modern Development of Unmusical Tone" (by Thos. C. Lewis).

### THE STUDENT OF MEDICINE.

### APPOINTMENTS.

M. L. B. Coombs, L.R.C.P., L.R.C.S.Edin., appointed Medical Officer of Health to the Newport Urban District, Isle of Wight. R. W. Councell, M.R.C.S.Eng., L.S.A., appointed Medical Officer for the Sixth District of the St. Saviour's Union. R. H. P. Crawfurd, M.A.Oxon., M.D., B.Ch., M.R.C.P.Lond., appointed Pathologist to the Royal Free Hospital, Gray's Inn Road. E. J. A. Dodd, M.R.C.S., L.R.C.P., appointed one of the House Surgeons to the Leeds General Infirmary. C. H. Fagge, M.R.C.S., L.R.C.P., appointed Surgical Registrar and Tutor to Guy's Hospital. J. Fawcett, M.D., B.S.Lond., F.R.C.S.Eng., appointed Assistant Physician to the Royal Free Hospital, Gray's Inn Road, W.C. J. M. Ferguson, L.R.C.P., L.R.C.S.Edin., appointed Medical Officer for the Third Burnley District of the Burnley Union. A. B. Franey, L.R.C.P., L.R.C.S.Edin., L.F.P.S.Glasg., appointed Medical Officer of Health to the Hadley Urban District Council. J. Harrison, M.R.C.S.Eng., L.R.C.P.Edin., appointed Medical Officer to the East Grinstead General Dispensary. J. V. Hartley, M.R.C.S., L.R.C.P., appointed Resident Medical Officer at the Ida Hospital of the Leeds General Infirmary. A. E. Hodges, L.R.C.P., L.R.C.S.Edin., appointed Medical Officer for the First Burnley District of the Burnley Union. J. A. Hope, M.B., C.M.Glas. appointed Assistant Medical Officer to the Barony Parish Hospital. E. J. Lumb, M.R.C.S., L.R.C.P., appointed one of the House Physicians to the Leeds General Infirmary. J. M. McGregor, M.B., C.M., appointed Clerk to the Extra Physicians and Registrar of the Royal Edinburgh Hospital for Sick Children. A. A. Martin, M.B., B.Sc.Lond., M.R.C.S., L.R.C.P., appointed House Physician to St. Mary's Hospital. F. W. Martin, M.R.C.S.Eng., L.R.C.P., L.R.C.S.Edin., appointed Medical Officer of Health to the Brighouse Town Council. D. Seaton, M.B., Ch.B., appointed Resident Surgical Officer to the Leeds General Infirmary. H. Silver, L.S.A., appointed Clinical Assistant to the Out-Patients at the Chelsea Hospital for Women, Fulham Road. H. Simpson, B.A.Cantab., M.B., B.C., M.R.C.S.Eng., appointed Medical Officer for the Market Weighton Second District of the Pocklington Union. M. F. Squire, M.B., B.S., M.R.C.S.Eng., L.R.C.P.Lond., appointed Assistant Medical Officer of the Workhouse of the Paddington Union. F. E. Taylor, M.A., B.Ch., M.B., appointed one of the House Surgeons to the Leeds General Infirmary. A. Ward, M.B., C.M.Edin., appointed Medical Officer for the Codford St. Peter District of the Warminster Union. J. C. Webster, M.D.Edin., F.R.C.P.E., F.R.S.E., late Senior Assistant to the Professor of Midwifery and Diseases of Women in the University of Edinburgh, appointed Assistant Gynæcologist to the Royal Victoria Hospital, Montreal.

